# Molecular Mechanisms Associated with ROR1-Mediated Drug Resistance: Crosstalk with Hippo-YAP/TAZ and BMI-1 Pathways

**DOI:** 10.3390/cells8080812

**Published:** 2019-08-02

**Authors:** Hanna Karvonen, Harlan Barker, Laura Kaleva, Wilhelmiina Niininen, Daniela Ungureanu

**Affiliations:** 1 Faculty of Medicine and Health Technology, Tampere University, 33014 Tampere, Finland; 2 Tays Cancer Center, Tampere University Hospital, 33520 Tampere, Finland

**Keywords:** Wnt signaling, ROR1, Wnt5a, Hippo-YAP/TAZ, BMI-1, drug resistance, cancer stem cells, stemness

## Abstract

Signaling via the Wnt-related receptor tyrosine kinase-like orphan receptor 1 (ROR1) triggers tumorigenic features associated with cancer stem cells (CSCs) and epithelial–mesenchymal transition (EMT), while aberrant expression of ROR1 is strongly linked to advanced disease progression and chemoresistance. Several recent studies have shown that Wnt5a binding to ROR1 promotes oncogenic signaling by activating multiple pathways such as RhoA/Rac1 GTPases and PI3K/AKT, which in turn could induce transcriptional coactivator YAP/TAZ or polycomb complex protein BMI-1 signaling, respectively, to sustain stemness, metastasis and ultimately drug-resistance. These data point towards a new feedback loop during cancer development, linking Wnt5a-ROR1 signaling activation to YAP/TAZ or BMI-1 upregulation that could play an important role in disease progression and treatment resistance. This review focuses on the crosstalk between Wnt5a-ROR1 and YAP/TAZ or the BMI-1 signaling network, together with the current advancements in targeted strategies for ROR1-positive cancers.

## 1. Introduction

The Wnt pathway plays essential roles in development, tissue homeostasis and disease, by transducing signals from 19 Wnt ligands via a complex network of receptors and co-receptors [1] that can be classified into canonical (β-catenin dependent) and non-canonical (β-catenin independent) Wnt signaling. Although there is a considerable overlap among Wnt ligands and their specific receptors when it comes to their involvement in canonical or non-canonical Wnt signaling, it is now accepted that the canonical Wnt pathway primarily regulates cell proliferation and differentiation, while non-canonical Wnt signaling regulates cell polarity, adhesion, and motility [2]. Dysregulation of Wnt signaling has been linked to a number of diseases, most notably cancer, along with other developmental, immunological and metabolic disorders [3].

Four Wnt binding receptors belong to the receptor tyrosine kinases as well as the pseudokinase family and are mediators of non-canonical Wnt signaling; these are receptor tyrosine kinase-like orphan receptor 1/2 (ROR1/ROR2), protein tyrosine kinase 7/colon carcinoma kinase 4 (PTK7/CCK4), and tyrosine protein kinase (RYK). The tyrosine kinase domains of these receptors lack several of the canonical amino acids or motifs that are typically required for proper catalytic activity and were unable to bind nucleotides in vitro, and are therefore considered to be “kinase dead” [4,5,6,7]. Despite their structural alterations and impaired catalytic activity, these Wnt receptor pseudokinases have been implicated in the pathology of several cancers and developmental disorders [8,9], via the modulation of their expression and crosstalk with various intracellular signaling networks.

ROR1 and ROR2 are the two members of the ROR receptor family that are strongly expressed during fetal and embryonic development, but to a lesser extent in tissues of healthy adults [10,11]. RORs are known to regulate several cellular processes including cell division, proliferation, migration, and chemotaxis, via the activation of planar cell polarity (PCP) and Ca^2+^ pathways from non-canonical Wnt signaling [1]. Both ROR receptors share a common molecular structure consisting of an extracellular domain (ECD) composed of an Ig-like, cysteine rich domain (CRD) and Kringle domain; a transmembrane domain; and an intracellular domain (ICD), consisting of a tyrosine kinase-like domain, followed by two serine/threonine domains flanking a proline-rich domain at the end of the ICD [10]. Wnt5a from the non-canonical Wnt pathway has been shown to bind to ROR1 and ROR2 and induce receptor heterodimerization, although more studies are needed on ROR homo- or heterodimerization mechanisms in different cellular models [12]. Moreover, other Wnt ligands have been shown to interact with either ROR1 or ROR2 in various cellular contexts, such as Wnt16–ROR1 interaction in TCF3-PBX1 B-cell acute lymphoblastic leukemia [13] and Wnt9a–ROR2 interaction during skeletal development [14].

**Figure 1:**
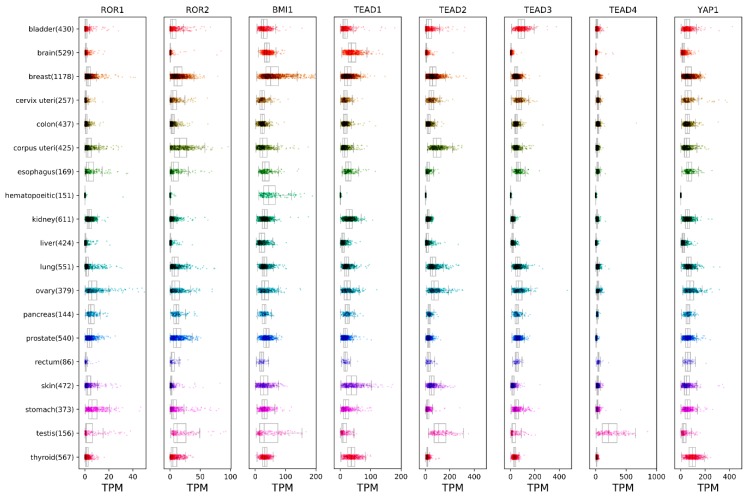
Expression analysis of receptor tyrosine kinase-like orphan receptor 1 (ROR1), ROR2, BMI-1 and YAP/TAZ genes in various human cancers. RNA-Seq expression data was obtained from The Cancer Genome Atlas (TCGA) Research Network data portal (https://portal.gdc.cancer.gov/). Fragments per kilobase million (FPKM) expression values were extracted for all target genes using custom Python scripts and converted to transcripts per million (TPM), and finally visualized as boxplots utilizing the Matplotlib [27] and Seaborn [28] plotting libraries.

ROR signaling has been implicated in the regulation of tissue development and homeostasis, as well as in tumorigenesis. The enhanced expression of ROR1 is associated with malignancies of several tissues, including hematological cancers such as chronic lymphocytic leukemia (CLL), mantle cell lymphoma (MCL), as well as ovarian, breast, prostate, lung, melanoma and colorectal cancers (Figure 1) [15]. The high expression of ROR1 in tumor cells is known to contribute to an enhanced rate of cell survival, proliferation, migration and chemotaxis. The binding of Wnt5a ligand leads to ROR1 receptor dimerization and the recruitment of various adaptor proteins that trigger the activation of downstream Rho/Rac1 GTPases [12,13,16], PI3K/AKT [17,18,19] and the Hippo-YAP/TAZ pathway [19,20,21], contributing to several biological processes which ultimately lead to tumor progression and chemoresistance. At the same time, elevated ROR2 expression has also been detected in many solid tumors including melanoma, breast, prostate, renal, colorectal, ovarian and endometrial cancers (Figure 1) [22,23,24]. High ROR2 expression is usually linked to the increased migration and invasiveness of cancer cells [22]. Additionally, ROR2 was recently found to be expressed in multiple myeloma, where it mediates the interaction between cancer cells and the bone marrow microenvironment niche [25]. Also, multipotent mesenchymal stem cells (MSCs) express ROR2 that enhances the chondrogenic potential of these cells, which could be useful in cartilage regeneration or production [26].

Metastasis and chemoresistance are the challenges of cancer therapy. Recently, strong evidence has associated ROR1 expression with cancer stem cells (CSCs), epithelial–mesenchymal transition (EMT) and chemoresistance, making this receptor a critical factor in tumor metastasis and recurrence [29,30]. Interestingly, in tumors such as breast cancer, enhanced ROR1 expression promotes tumorigenesis through the upregulation of YAP/TAZ transcription and/or polycomb complex protein BMI-1 expression. This indicates new crosstalk by which the non-canonical Wnt pathway, via Wnt5a-ROR1 engagement, could sustain malignant transformation [19,20,21,31]. This is important as YAP/TAZ transcription factors are both frequently altered in many types of cancers [32,33], while elevated BMI-1 was also reported to be important for tumorigenesis [34]. The Wnt5a-ROR1-dependent upregulation of YAP/TAZ and BMI-1 points toward a new positive feedback loop that may play an important role in mediating cancer stemness, tumorigenesis and drug resistance.

Recent clinical strategies have focused on the direct targeting of ROR1 using monoclonal antibodies (mAbs). Many ROR1 mAbs have been designed and demonstrate significant preclinical efficacy [35,36,37]; cirmtuzumab (UC-951) [38] is currently in phase I–II of clinical trials for CLL and breast cancer. Encouraging preclinical studies suggest that the concomitant suppression of ROR1 and other oncogenic pathways may be helpful in attaining greater clinical benefits [13,20,39,40]. This review focuses on the latest advances in our understanding regarding the crosstalk of Wnt5a-ROR1 and Hippo-YAP/TAZ or BMI-1 in tumorigenesis and drug resistance and discusses novel strategies for the therapeutic targeting of ROR1 in cancer treatment.

## 2. ROR1 Expression Is Linked to the CSC Phenotype and the Development of Chemoresistance

The existence of the cancer stem cell (CSC) has emerged recently as increasingly relevant in regards to tumor development, metastasis, recurrence and drug resistance [41]. CSCs are defined as a subpopulation of cancer cells endowed with self-renewal and differentiation properties, through stemness pathways, such as Wnt, TGF-β, STAT, and Hippo-YAP/TAZ, among others. Although their origin remains elusive [42], CSCs are important contributors to intratumoral heterogeneity and are strongly associated with disease recurrence and treatment resistance [43]. The identification of several CSC markers has facilitated the screening and study of many cancers, including those of the blood, breast, ovaries, brain, lung, colon and skin [44]. Currently, CD133, CD24, CD44 and aldehyde dehydrogenase 1 (ALDH1) are widely used to identify CSCs in various tumors, while other markers such as BMI-1 [45] and ROR1 [20,46,47] are starting to gain more attention for their involvement in the stemness of several malignancies. ROR1 has been associated with the CSC phenotype in several malignancies such as leukemia, ovarian and breast tumors. The majority of CLL patients express ROR1 on their neoplastic B cells that possess stemness properties such as self-renewal and differentiation [38], while the expression of ROR1 in mouse models of CLL demonstrated the activation of signaling networks implicated in CSC self-renewal [48]. Furthermore, high ROR1 levels in CLL patient samples correlated with relatively short overall survival rates. Further evidence for ROR1 involvement in CSCs has emerged from studies of ovarian and breast cancers. Ovarian cancers with high levels of ROR1 have stem cell-like gene expression signatures, as demonstrated by high levels of ALDH1 [47] or the cell surface expression of CD133 and CD44 [49]. Moreover, ROR1-positive ovarian CSCs have an enhanced ability to seed metastasis, form tumor spheroids, engraft immune-deficient mice, and migrate better, making ROR1 a prognostic marker for the shorter overall survival of ovarian cancer patients [50]. Similarly, breast cancer cells with high ROR1 expression rates have elevated levels of stemness signature genes (*Nanog*, *Oct4*, and *Sox2*) and an increased ability to migrate and form spheroids or engraftments in immune-deficient mice, strongly indicating that ROR1 could be a marker for CSCs in breast tumors [20]. Conclusively, these findings emphasize a key functional role for ROR1 that could contribute to the biological phenotypes associated with CSCs.

High levels of ROR1 expression in various malignancies are functionally relevant to mediate drug resistance and are usually associated with advanced disease stages. For example, ovarian and breast cancer tumors with high levels of ROR1 were typically poorly differentiated and associated with an aggressive disease stage [20,51]. Recent studies have also shown that acquired resistance to T-DM1 (trastuzumab emtansine, a newly developed antibody-drug conjugate) in HER2^+^ breast cancer patients induced high ROR1 expression on neoplastic cells that exhibit stem cell signatures (such as expression of both CD44 and ALDH1), resulting in an enhanced capacity to form spheroids and self-renewal properties [21]. In another disease model, resistance to the MEK inhibitor trametinib in uveal melanoma was associated with the upregulation of ROR1 and YAP/TAZ signaling, resulting in treatment resistance in preclinical models. The inhibition of the MEK pathway led to an increase in AKT phosphorylation, which was partially due to the Wnt5a-mediated activation of ROR1/ROR2, while the subsequent silencing of RORs resulted in a decreased AKT phosphorylation. On the other hand, trametinib treatment led to a strong induction of G-protein coupled receptors (GPCRs) that in turn upregulated YAP signaling via a release of ET-3 (endotelin-3)—in this way, YAP is activated in an autocrine fashion through EDNRB (endothelin receptor B) [19]. This is a good example of how cancer cells could employ several “escape” routes to overcome treatment inhibition and ultimately develop resistance.

Interestingly, a recent transcriptomic and proteomic analysis of breast cancer models showed that the stress hormone pathway effectively induces metastasis development with a distinct phenotype [52]. High GC (glucocorticoid) levels increased tumor heterogeneity and metastasis by upregulating some of the non-canonical Wnt pathway genes, highlighted by ROR1 expression. A transcriptome analysis of breast cancer metastasis indicated a direct correlation between ROR1 levels and GR (glucocorticoid receptor) signaling activation. GR activation increased metastatic breast cancer development in a ROR1-dependent manner, while decreasing the efficacy of paclitaxel. Cancer cells with upregulated ROR1 could be a good opportunity for targeted therapies, where the deleterious effect of GR activation in stress hormone-induced metastasis is prevented by ROR1 inhibition, resulting in cancer cell death.

Given the above examples, it is now more clear that ROR1 expression triggers disease progression and drug resistance via CSC development, suggesting that ROR1 could also be used as a CSC marker, at least for some tumors such as breast and ovarian cancers.

## 3. Wnt5a-ROR1 Signaling Upregulates BMI-1 to Sustain Stemness and Drug Resistance

ROR1 signaling that contributes to the maintenance, self-renewal, and drug resistance of CSCs has been uncovered for some cancers. In breast cancer, Wnt5a binding to ROR1 increases BMI-1 levels and this process was dependent on AKT phosphorylation [20]. The knockdown of ROR1 expression or the blocking of ROR1 signaling with cirmtuzumab impaired Wnt5a-mediated AKT phosphorylation and BMI-1 accumulation, clearly indicating that the intracellular signaling downstream ROR1 is involved in BMI-1 expression modulation. Interestingly, Wnt5a-mediated BMI-1 upregulation in breast cancer cells was observed only at the protein level and not at the mRNA level, indicating that this process is not regulated via transcriptional activation. Previous reports have shown that phosphorylated AKT could elevate BMI-1 levels to modulate its oncogenic function [53]. Indeed, blocking the AKT phosphorylation downstream Wnt5a-ROR1 signaling activation resulted in a loss of BMI-1 upregulation, indicating that the activation of AKT is the switch for BMI-1 expression modulation.

Similar findings have been described in the T-DM1-induced drug resistance of breast cancer cells, which resulted in elevated ROR1 levels [21]. Consequently, cancer cells expressing ROR1 at higher levels had increased BMI-1 expression and stemness properties, as demonstrated by elevated *Nanog, Oct3/4* and *Sox2* expression.

**Figure 2:**
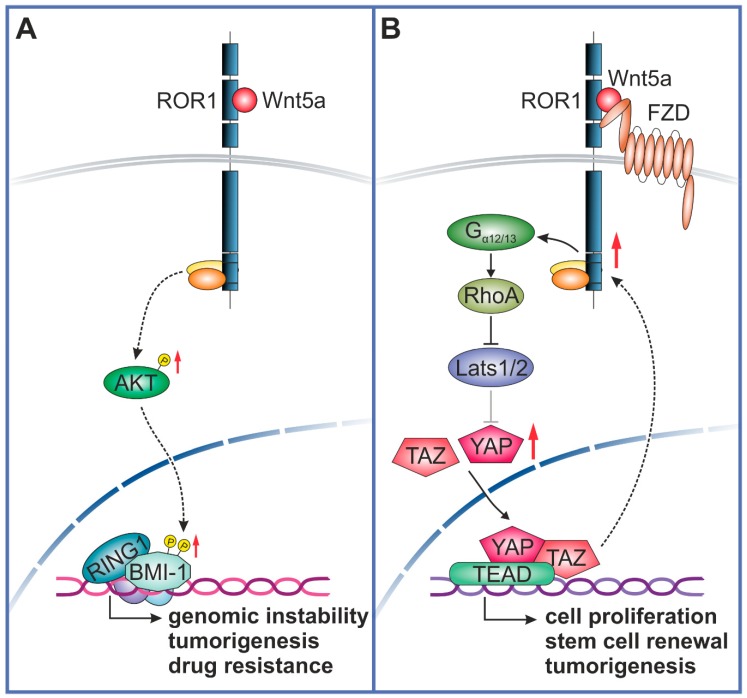
Crosstalk between Wnt5a-ROR1 signaling and BMI-1 (**A**) or YAP/TAZ (**B**) pathways. (**A**) Wnt5a binding to ROR1 induces intracellular signaling, leading to an increased AKT activation that in turn can phosphorylate BMI-1 to promote genomic instability, cancer cell proliferation and drug-resistance. (**B**) Wnt5a binding to ROR1/FZD complex will activate RhoA via G_α12/13_ engagement, resulting in the inhibition of Lats1/2 activity and consequently, YAP/TAZ dephosphorylation and nuclear translocation. Binding of YAP/TAZ to TEADs can induce the transcription of genes involved in stemness and tumorigenesis. Increased YAP/TAZ transcription could, in turn, upregulate ROR1 expression. RingB1, E3 ubiquitin-protein ligase RING1; FZD, Frizzled; G_α12/13_, Guanine nucleotide-binding protein subunit alpha-12/13; AKT, protein kinase B.

BMI-1 is a member of the polycomb repressive complex 1 (PRC1) that functions as an epigenetic suppressor of gene expression via heterodimerization with Ring1B/Rnf2. Ring1B/Rnf2 heterodimer stimulates BMI-1 ubiquitin ligase activity toward lysine 119 of histone 2A (H2A-K119) [54]. BMI-1 is recruited to CpG islands throughout the genome by transcription factors and non-coding RNAs [55] to mediate gene silencing. Although it is well known that BMI-1 has an important role in the modulation of self-renewal and cell differentiation potential of hematopoietic stem cells (HSCs) and neuronal stem cells (NSCs) [34], BMI-1 is also a key regulator of the self-renewal, differentiation and tumor initiation of CSCs. BMI-1 is expressed in a number of malignancies, such as prostate, lung, ovarian, pancreatic, lymphoma, glioma, acute myeloid leukemia and breast cancer, among others [34] (Figure 1 [27,28]). High levels of BMI-1 in tumor cells drive stem-like properties associated with the induction of EMT that promotes invasion, metastasis, and an enhanced resistance to chemotherapy [56,57,58]. For instance, in breast cancer, the knockdown of BMI-1 expression impaired the tumor initiation ability of CSCs, while the rescue of BMI-1 expression restored tumor formation [59]. Moreover, high levels of BMI-1 were found in human glioblastomas, specifically in the tumor-initiating CD133 positive stem cells, and the knockdown of BMI-1 completely prevented brain tumor formation in mice [60]. Enhanced BMI-1 levels upon Wnt5a-ROR1 stimulation via AKT activation may indicate the existence of a crosstalk between non-canonical Wnt pathways and BMI-1, that could regulate tumorigenesis and drug resistance (Figure 2A). Prior reports have demonstrated a complex regulation of BMI-1 activation via phosphorylation, since AKT-mediated phosphorylation of BMI-1 could modulate its function in a Ink4a/Arf-independent [53] or dependent manner [61], to make BMI-1 an oncogene or a tumor suppressor gene, respectively, with opposing effects on cancer outcome. Given the above evidence, it appears that Wnt5a-ROR1 signaling could induce stemness and tumorigenesis via BMI-1 expression modulation, although further studies are needed to carefully delineate the phosphorylation-dependent regulation of the BMI-1 function downstream of the Wnt5a-ROR1 pathway.

## 4. Crosstalk between Wnt5a-ROR1 and YAP/TAZ Pathways in Tumorigenesis

Several studies have linked the expression of ROR1 to the activation of YAP/TAZ transcription, leading to enhanced tumorigenesis and chemoresistance [20,21]. YAP and TAZ are transcriptional effectors of the Hippo pathway, a key regulator of development and tissue homeostasis [62]. The core Mammalian Ste20-like kinase 1/2-Large tumor suppressor 1/2 (Mst1/2-Lats1/2) kinase cascade from the Hippo pathway controls the turnover of YAP/TAZ proteins via phosphorylation and ubiquitin-mediated degradation in the cytoplasm. Upon inhibition of the Hippo pathway, activated YAP/TAZ are translocated into the nucleus to bind TEAD family transcription factors and subsequently initiate the expression of target genes involved in cell proliferation, stem cell self-renewal, and tumorigenesis [63]. The dysregulation of Hippo signaling is observed in many cancers and can cause YAP/TAZ oncogenic addiction, resulting in stemness properties, proliferation, metastasis and drug-resistance [63]. For instance, TAZ activity is increased in poorly differentiated breast cancer tumors and correlates with CSCs, poor prognosis and metastasis [64], making TAZ an effector for self-renewal and tumor initiation properties of breast CSCs. Furthermore, in lung cancer, TAZ promotes tumorigenesis and stemness via the upregulation of the CSC marker ALDH1A [65]. Elevated YAP expression has also been observed in lung, liver, ovary, cervix, colon, and other cancers [32,33] (Figure 1 [27,28]).

Wnt5a stimulation induces the ROR1-dependent activation and nuclear accumulation of the TAZ protein, thereby enhancing the stemness of breast cancer cells, resulting in improved cell survival, migration and drug resistance [20]. There could be various molecular mechanisms responsible for the Wnt5a-ROR1-mediated YAP/TAZ transcription activation. Prior studies have indicated that Wnt5a/b ligands are able to activate YAP/TAZ via ROR1-FZD2/5 co-receptors through non-canonical Wnt signaling [66]. Also, YAP expression significantly increased the transcription of Wnt5a only, among other Wnt ligands, strongly indicating that YAP/TAZ are modulators of the non-canonical Wnt pathway via the induction of Wnt5a transcription. The molecular effectors directly connecting Wnt signaling to YAP/TAZ have been delineated to involve GPCR (G protein-coupled receptors) Gα_12/13_ and their downstream Rho GTPases that are directly acting on Lats1/2 activation and thereby, YAP/TAZ turnover (Figure 2). Prior studies have also shown that RhoA inhibits Lats1/2 to activate YAP/TAZ, while analysis of gene expression data from cancer cell lines revealed a direct correlation between YAP/TAZ and Wnt5a/b levels, indicating that non-canonical Wnt signaling via Wnt5a/b is able to induce YAP/TAZ activation in cancer cells [67].

Further evidence linking the ROR1 and YAP/TAZ pathways to the modulation of CSC self-renewal and drug resistance was provided recently by a study of T-MD1 resistance development in breast cancer [21]. T-DM1 resistance resulted in increased cell surface levels of ROR1 along with CSC-like properties, such as higher percentages of ALDH1+ and CD44+ in ROR1+/T-DM1-resistant breast cancer cells. Interestingly, YAP1 was found to regulate ROR1 expression, and the disruption of YAP1-TEAD impaired T-DM1-induced ROR1 overexpression and CSC self-renewal.

Another possible link between Hippo-YAP/TAZ and ROR1 signaling is the transcription factor Twist1, which is known to promote EMT in cancer cells. Twist1 expression is regulated by YAP in association with Smad2/3 [68]. Moreover, Twist1 has been shown to bind directly to the ROR1 promoter and induce ROR1 expression in basal-like breast cancer [69], as well as to regulate the transcription of ROR1 ligand Wnt5a [70].

Interestingly, aberrant YAP/TAZ expression has been linked to drug resistance in several cancers, underlining a common feature with ROR1 and raising the possibility that ROR1 and YAP/TAZ signaling reinforce each other to make cancer cells treatment resistant. YAP/TAZ overexpression could induce resistance to taxanes and doxorubicin in breast cancer [71,72]. In taxane-resistant ovarian cancer, YAP is activated as a result of miRNA targeting the mRNA transcription of Lats, an upstream deactivator of YAP/TAZ [72]. Other studies have reported that increased nuclear YAP levels could promote cisplatin resistance in ovarian cancer, squamous cell carcinoma, and urothelial cell carcinoma [71,73]. Moreover, the transcriptional targets of YAP/TAZ, such as CTGF and CYR61, can contribute to chemoresistance by activating the MAPK and NF-κB signaling pathways [72]. Additionally, YAP/TAZ/TEAD complexes induce the expression of ABC multidrug transporters that promote drug efflux; ABCB1 and ABCC1 have been found upregulated in ovarian and liver CSCs, and ABCG2 in gastric CSCs [72].

Taken together, these studies demonstrate the existence of a Wnt5a/ROR1–YAP/TAZ feedback loop that modulates the CSC phenotype, tumor progression, metastasis and, ultimately, drug resistance. The ROR1-dependent regulation of BMI-1 levels constitutes another signaling niche responsible for stemness, metastasis and drug resistance. Therefore, a better understanding of the crosstalk between the non-canonical Wnt pathway (Wnt5a/ROR1) and YAP/TAZ transcription or BMI-1 functional modulation would enable us to find more optimal therapeutic targets to overcome disease progression and treatment resistance.

## 5. Progress in ROR1-Targeted Therapies

The emergence of ROR1 as a marker for CSCs implies that ROR1-positive tumors have a high resistance to treatment. Indeed, ROR1-positive carcinomas were associated with aggressive disease progression and lower rates of progression-free survival or overall survival rates of patients, as well as treatment resistance, suggesting a high potential for ROR1-targeted therapies during advanced disease stages. The extracellular domain of ROR1 is typically targeted using antibody-based agents, and several of such antibodies have shown promising results in preclinical or clinical settings. Anti-ROR1 monoclonal antibody cirmtuzumab (UC-961) has completed a phase 1 clinical trial for CLL with encouraging results, as demonstrated by the inhibition of Wnt5a/ROR1 signaling in ROR1-positive leukemic cells, and a phase 1b/2 combination clinical trial has been initiated for patients with CLL and MCL [38]. Multiple preclinical studies have indicated that combination therapies co-targeting ROR1 and other oncogenic pathways are more effective in killing drug-resistant cancer cells than a single agent alone. For instance, co-treatment of breast cancer PDXs with cirmtuzumab and paclitaxel achieved a greater cytotoxic effect than a single treatment [20]. In similar studies for hematological cancers, the combination of cirmtuzumab and ibrutinib was more efficient in eradicating leukemic cells in vivo [74], whereas the ex vivo co-targeting of ROR1 and Bcl-2 in CLL, MCL or B-ALL patient samples was significantly more effective than targeting a single pathway, strongly supporting the implementation of these combinatorial regimens in clinical trials [13,39,40]. Apart from cirmtuzumab, other ROR1 monoclonal antibodies have been developed and tested at preclinical levels [75]. A potent immunotherapeutic approach was used to develop bispecific antibodies (biAbs) that combine a T cell-engaging arm with a tumor cell-binding arm, for which ROR1 seemed a perfect candidate due to its relatively restricted expression on tumor cells. A ROR1-targeting scFv with a membrane-proximal epitope, R11, which binds to the Kringle domain of ROR1, showed promising in vivo and ex vivo cytotoxicity against ROR1-positive cancer cells [76].

Moreover, ARI-1 ((R)-5,7-bis(methoxymethoxy)-2-(4-methoxyphenyl)chroman-4-one) inhibitor, targeting the extracellular Frizzled-like domain (CRD or cysteine-rich domain) of ROR1, produced promising results in chemoresistant NSCLC models [77]. Targeting the intracellular domains of ROR1 has posed more challenges, due to the lack of structural data for the cytoplasmic portion of this receptor. We therefore believe that more opportunities will present themselves once there are advances in understating the structural and functional aspects of ROR1 signaling mechanisms. This will have great implications for ROR1-targeted therapies, as well for the basic research of Wnt-signaling in development and disease.

## References

[1] Niehrs C. (2012). The complex world of WNT receptor signalling. Nat. Rev. Mol. Cell Biol..

[2] van Amerongen R., Nusse R. (2009). Towards an integrated view of Wnt signaling in development. Dev. Camb. Engl..

[3] Zimmerman Z.F., Moon R.T., Chien A.J. (2012). Targeting Wnt Pathways in Disease. Cold Spring Harb. Perspect. Biol..

[4] Mendrola J.M., Shi F., Park J.H., Lemmon M.A. (2013). Receptor Tyrosine Kinases with Intracellular Pseudokinase Domains. Biochem. Soc. Trans..

[5] Murphy J.M., Zhang Q., Young S.N., Reese M.L., Bailey F.P., Eyers P.A., Ungureanu D., Hammaren H., Silvennoinen O., Varghese L.N. (2014). A robust methodology to subclassify pseudokinases based on their nucleotide-binding properties. Biochem. J..

[6] Eyers P.A., Murphy J.M. (2013). Dawn of the dead: Protein pseudokinases signal new adventures in cell biology. Biochem. Soc. Trans..

[7] Byrne D.P., Foulkes D.M., Eyers P.A. (2017). Pseudokinases: Update on their functions and evaluation as new drug targets. Future Med. Chem..

[8] Kung J.E., Jura N. (2019). Prospects for pharmacological targeting of pseudokinases. Nat. Rev. Drug Discov..

[9] Al-Shawi R., Ashton S.V., Underwood C., Simons J.P. (2001). Expression of the Ror1 and Ror2 receptor tyrosine kinase genes during mouse development. Dev. Genes Evol..

[10] Karvonen H., Niininen W., Murumägi A., Ungureanu D. (2017). Targeting ROR1 identifies new treatment strategies in hematological cancers. Biochem. Soc. Trans..

[11] Borcherding N., Kusner D., Liu G.-H., Zhang W. (2014). ROR1, an embryonic protein with an emerging role in cancer biology. Protein Cell.

[12] Yu J., Chen L., Cui B., Widhopf G.F., Shen Z., Wu R., Zhang L., Zhang S., Briggs S.P., Kipps T.J. (2016). Wnt5a induces ROR1/ROR2 heterooligomerization to enhance leukemia chemotaxis and proliferation. J. Clin. Invest..

[13] Karvonen H., Perttilä R., Niininen W., Hautanen V., Barker H., Murumägi A., Heckman C.A., Ungureanu D. (2019). Wnt5a and ROR1 activate non-canonical Wnt signaling via RhoA in TCF3-PBX1 acute lymphoblastic leukemia and highlight new treatment strategies via Bcl-2 co-targeting. Oncogene.

[14] Weissenböck M., Latham R., Nishita M., Wolff L.I., Ho H.-Y.H., Minami Y., Hartmann C. (2019). Genetic interactions between Ror2 and Wnt9a, Ror1 and Wnt9a and Ror2 and Ror1: Phenotypic analysis of the limb skeleton and palate in compound mutants. Genes Cells.

[15] Saleh R.R., Antrás J.F., Peinado P., Pérez-Segura P., Pandiella A., Amir E., Ocaña A. (2019). Prognostic value of receptor tyrosine kinase-like orphan receptor (ROR) family in cancer: A meta-analysis. Cancer Treat. Rev..

[16] Wu X., Yan T., Hao L., Zhu Y. (2019). Wnt5a induces ROR1 and ROR2 to activate RhoA in esophageal squamous cell carcinoma cells. Cancer Manag. Res..

[17] Cui B., Ghia E.M., Chen L., Rassenti L.Z., DeBoever C., Widhopf G.F., Yu J., Neuberg D.S., Wierda W.G., Rai K.R. (2016). High-level ROR1 associates with accelerated disease progression in chronic lymphocytic leukemia. Blood.

[18] Yamaguchi T., Lu C., Ida L., Yanagisawa K., Usukura J., Cheng J., Hotta N., Shimada Y., Isomura H., Suzuki M. (2016). ROR1 sustains caveolae and survival signalling as a scaffold of cavin-1 and caveolin-1. Nat. Commun..

[19] Faião-Flores F., Emmons M.F., Durante M.A., Kinose F., Saha B., Fang B., Koomen J.M., Chellappan S.P., Maria-Engler S.S., Rix U. (2019). HDAC inhibition enhances the in vivo efficacy of MEK inhibitor therapy in uveal melanoma. Clin. Cancer Res. Off. J. Am. Assoc. Cancer Res..

[20] Zhang S., Zhang H., Ghia E.M., Huang J., Wu L., Zhang J., Lam S., Lei Y., He J., Cui B. (2019). Inhibition of chemotherapy resistant breast cancer stem cells by a ROR1 specific antibody. Proc. Natl. Acad. Sci. USA.

[21] Islam S.S., Uddin M., Noman A.S.M., Akter H., Dity N.J., Basiruzzman M., Uddin F., Ahsan J., Annoor S., Alaiya A.A. (2019). Antibody-drug conjugate T-DM1 treatment for HER2+ breast cancer induces ROR1 and confers resistance through activation of Hippo transcriptional coactivator YAP1. EBioMedicine.

[22] Debebe Z., Rathmell W.K. (2015). Ror2 as a Therapeutic Target in Cancer. Pharmacol. Ther..

[23] Henry C., Llamosas E., Knipprath-Meszaros A., Schoetzau A., Obermann E., Fuenfschilling M., Caduff R., Fink D., Hacker N., Ward R. (2015). Targeting the ROR1 and ROR2 receptors in epithelial ovarian cancer inhibits cell migration and invasion. Oncotarget.

[24] Henry C.E., Llamosas E., Daniels B., Coopes A., Tang K., Ford C.E. (2018). ROR1 and ROR2 play distinct and opposing roles in endometrial cancer. Gynecol. Oncol..

[25] Frenquelli M., Caridi N., Antonini E., Storti F., Viganò V., Gaviraghi M., Occhionorelli M., Bianchessi S., Bongiovanni L., Spinelli A. (2019). The WNT receptor ROR2 drives the interaction of multiple myeloma cells with the microenvironment through AKT activation. Leukemia.

[26] Dickinson S.C., Sutton C.A., Brady K., Salerno A., Katopodi T., Williams R.L., West C.C., Evseenko D., Wu L., Pang S. (2017). The Wnt5a Receptor, Receptor Tyrosine Kinase-Like Orphan Receptor 2, Is a Predictive Cell Surface Marker of Human Mesenchymal Stem Cells with an Enhanced Capacity for Chondrogenic Differentiation. Stem Cells Dayt. Ohio.

[27] Hunter J.D. (2007). Matplotlib: A 2D Graphics Environment. Comput. Sci. Eng..

[28] Waskom M., Botvinnik O., O’Kane D., Hobson P., Lukauskas S., Gemperline D.C., Augspurger T., Halchenko Y., Cole J.B., Warmenhoven J. mwaskom/seaborn: v0.8.1 (September 2017).

[29] Katoh M. (2017). Canonical and non-canonical WNT signaling in cancer stem cells and their niches: Cellular heterogeneity, omics reprogramming, targeted therapy and tumor plasticity (Review). Int. J. Oncol..

[30] Cui B., Zhang S., Chen L., Yu J., Widhopf G.F., Fecteau J.-F., Rassenti L.Z., Kipps T.J. (2013). Targeting ROR1 inhibits epithelial-mesenchymal transition and metastasis. Cancer Res..

[31] Katoh M., Katoh M. (2017). Molecular genetics and targeted therapy of WNT-related human diseases (Review). Int. J. Mol. Med..

[32] He C., Mao D., Hua G., Lv X., Chen X., Angeletti P.C., Dong J., Remmenga S.W., Rodabaugh K.J., Zhou J. (2015). The Hippo/YAP pathway interacts with EGFR signaling and HPV oncoproteins to regulate cervical cancer progression. EMBO Mol. Med..

[33] He C., Lv X., Huang C., Hua G., Ma B., Chen X., Angeletti P.C., Dong J., Zhou J., Wang Z. (2019). YAP1-LATS2 feedback loop dictates senescent or malignant cell fate to maintain tissue homeostasis. EMBO Rep..

[34] Bhattacharya R., Banerjee Mustafi S., Street M., Dey A., Dwivedi S.K.D. (2015). Bmi-1: At the crossroads of physiological and pathological biology. Genes Dis..

[35] Aghebati-Maleki L., Younesi V., Baradaran B., Abdolalizadeh J., Motallebnezhad M., Nickho H., Shanehbandi D., Majidi J., Yousefi M. (2017). Antiproliferative and Apoptotic Effects of Novel Anti-ROR1 Single-Chain Antibodies in Hematological Malignancies. SLAS Discov. Adv. Life Sci. R D.

[36] Aghebati-Maleki L., Younesi V., Jadidi-Niaragh F., Baradaran B., Majidi J., Yousefi M. (2017). Isolation and characterization of anti ROR1 single chain fragment variable antibodies using phage display technique. Hum. Antibodies.

[37] Hassannia H., Amiri M.M., Jadidi-Niaragh F., Hosseini-Ghatar R., Khoshnoodi J., Sharifian R.-A., Golsaz-Shirazi F., Jeddi-Tehrani M., Shokri F. (2018). Inhibition of tumor growth by mouse ROR1 specific antibody in a syngeneic mouse tumor model. Immunol. Lett..

[38] Choi M.Y., Widhopf G.F., Ghia E.M., Kidwell R.L., Hasan M.K., Yu J., Rassenti L.Z., Chen L., Chen Y., Pittman E. (2018). Phase I Trial: Cirmtuzumab Inhibits ROR1 Signaling and Stemness Signatures in Patients with Chronic Lymphocytic Leukemia. Cell Stem Cell..

[39] Karvonen H., Chiron D., Niininen W., Ek S., Jerkeman M., Moradi E., Nykter M., Heckman C.A., Kallioniemi O., Murumägi A. (2017). Crosstalk between ROR1 and BCR pathways defines novel treatment strategies in mantle cell lymphoma. Blood Adv..

[40] Rassenti L.Z., Balatti V., Ghia E.M., Palamarchuk A., Tomasello L., Fadda P., Pekarsky Y., Widhopf G.F., Kipps T.J., Croce C.M. (2017). MicroRNA dysregulation to identify therapeutic target combinations for chronic lymphocytic leukemia. Proc. Natl. Acad. Sci..

[41] Yu Z., Pestell T.G., Lisanti M.P., Pestell R.G. (2012). Cancer Stem Cells. Int. J. Biochem. Cell Biol..

[42] Dawood S., Austin L., Cristofanilli M. (2014). Cancer stem cells: Implications for cancer therapy. Oncol. Williston Park N.

[43] Hanahan D., Weinberg R.A. (2011). Hallmarks of cancer: The next generation. Cell.

[44] Ajani J.A., Song S., Hochster H.S., Steinberg I.B. (2015). Cancer stem cells: The promise and the potential. Semin. Oncol..

[45] Cao L., Bombard J., Cintron K., Sheedy J., Weetall M.L., Davis T.W. (2011). BMI1 as a novel target for drug discovery in cancer. J. Cell. Biochem..

[46] Wu D., Yu X., Wang J., Hui X., Zhang Y., Cai Y., Ren M., Guo M., Zhao F., Dou J. (2019). Ovarian Cancer Stem Cells with High ROR1 Expression Serve as a New Prophylactic Vaccine for Ovarian Cancer. J. Immunol. Res..

[47] Zhang S., Cui B., Lai H., Liu G., Ghia E.M., Widhopf G.F., Zhang Z., Wu C.C.N., Chen L., Wu R. (2014). Ovarian cancer stem cells express ROR1, which can be targeted for anti-cancer-stem-cell therapy. Proc. Natl. Acad. Sci. USA.

[48] Widhopf G.F., Cui B., Ghia E.M., Chen L., Messer K., Shen Z., Briggs S.P., Croce C.M., Kipps T.J. (2014). ROR1 can interact with TCL1 and enhance leukemogenesis in Eµ-TCL1 transgenic mice. Proc. Natl. Acad. Sci..

[49] Gonzalez V.D., Samusik N., Chen T.J., Savig E.S., Aghaeepour N., Quigley D.A., Huang Y.-W., Giangarrà V., Borowsky A.D., Hubbard N.E. (2018). Commonly Occurring Cell Subsets in High-Grade Serous Ovarian Tumors Identified by Single-Cell Mass Cytometry. Cell Rep..

[50] Zhang H., Qiu J., Ye C., Yang D., Gao L., Su Y., Tang X., Xu N., Zhang D., Xiong L. (2014). ROR1 expression correlated with poor clinical outcome in human ovarian cancer. Sci. Rep..

[51] Winter W.E., Maxwell G.L., Tian C., Carlson J.W., Ozols R.F., Rose P.G., Markman M., Armstrong D.K., Muggia F., McGuire W.P. (2007). Prognostic factors for stage III epithelial ovarian cancer: A Gynecologic Oncology Group Study. J. Clin. Oncol. Off. J. Am. Soc. Clin. Oncol..

[52] Obradović M.M.S., Hamelin B., Manevski N., Couto J.P., Sethi A., Coissieux M.-M., Münst S., Okamoto R., Kohler H., Schmidt A. (2019). Glucocorticoids promote breast cancer metastasis. Nature.

[53] Nacerddine K., Beaudry J.-B., Ginjala V., Westerman B., Mattiroli F., Song J.-Y., van der Poel H., Ponz O.B., Pritchard C., Cornelissen-Steijger P. (2012). Akt-mediated phosphorylation of Bmi1 modulates its oncogenic potential, E3 ligase activity, and DNA damage repair activity in mouse prostate cancer. J. Clin. Invest..

[54] Buchwald G., van der Stoop P., Weichenrieder O., Perrakis A., van Lohuizen M., Sixma T.K. (2006). Structure and E3-ligase activity of the Ring-Ring complex of polycomb proteins Bmi1 and Ring1b. EMBO J..

[55] Blackledge N.P., Rose N.R., Klose R.J. (2015). Targeting Polycomb systems to regulate gene expression: Modifications to a complex story. Nat. Rev. Mol. Cell Biol..

[56] Grinstein E., Mahotka C. (2009). Stem cell divisions controlled by the proto-oncogene BMI-1. J. Stem Cells.

[57] Lukacs R.U., Memarzadeh S., Wu H., Witte O.N. (2010). Bmi-1 is a crucial regulator of prostate stem cell self-renewal and malignant transformation. Cell Stem Cell.

[58] Wu X., Liu X., Sengupta J., Bu Y., Yi F., Wang C., Shi Y., Zhu Y., Jiao Q., Song F. (2011). Silencing of Bmi-1 gene by RNA interference enhances sensitivity to doxorubicin in breast cancer cells. Indian, J. Exp. Biol..

[59] Wang M.-C., Li C.-L., Cui J., Jiao M., Wu T., Jing L., Nan K.-J. (2015). BMI-1, a promising therapeutic target for human cancer. Oncol. Lett..

[60] Abdouh M., Facchino S., Chatoo W., Balasingam V., Ferreira J., Bernier G. (2009). BMI1 sustains human glioblastoma multiforme stem cell renewal. J. Neurosci. Off. J. Soc. Neurosci..

[61] Liu Y., Liu F., Yu H., Zhao X., Sashida G., Deblasio A., Harr M., She Q.-B., Chen Z., Lin H.-K. (2012). Akt phosphorylates the transcriptional repressor bmi1 to block its effects on the tumor-suppressing ink4a-arf locus. Sci. Signal..

[62] Snigdha K., Gangwani K.S., Lapalikar G.V., Singh A., Kango-Singh M. (2019). Hippo Signaling in Cancer: Lessons From Drosophila Models. Front. Cell Dev. Biol..

[63] Mo J.-S., Park H.W., Guan K.-L. (2014). The Hippo signaling pathway in stem cell biology and cancer. EMBO Rep..

[64] Cordenonsi M., Zanconato F., Azzolin L., Forcato M., Rosato A., Frasson C., Inui M., Montagner M., Parenti A.R., Poletti A. (2011). The Hippo Transducer TAZ Confers Cancer Stem Cell-Related Traits on Breast Cancer Cells. Cell.

[65] Lo Sardo F., Strano S., Blandino G. (2018). YAP and TAZ in Lung Cancer: Oncogenic Role and Clinical Targeting. Cancers.

[66] Park H.W., Kim Y.C., Yu B., Moroishi T., Mo J.-S., Plouffe S.W., Meng Z., Lin K.C., Yu F.-X., Alexander C.M. (2015). Alternative Wnt Signaling Activates YAP/TAZ. Cell.

[67] Yu F.-X., Zhao B., Panupinthu N., Jewell J.L., Lian I., Wang L.H., Zhao J., Yuan H., Tumaneng K., Li H. (2012). Regulation of the Hippo-YAP Pathway by G-Protein-Coupled Receptor Signaling. Cell.

[68] Zhang H., von Gise A., Liu Q., Hu T., Tian X., He L., Pu W., Huang X., He L., Cai C.-L. (2014). Yap1 Is Required for Endothelial to Mesenchymal Transition of the Atrioventricular Cushion. J. Biol. Chem..

[69] Cao J., Wang X., Dai T., Wu Y., Zhang M., Cao R., Zhang R., Wang G., Jiang R., Zhou B.P. (2018). Twist promotes tumor metastasis in basal-like breast cancer by transcriptionally upregulating ROR1. Theranostics.

[70] Shi J., Wang Y., Zeng L., Wu Y., Deng J., Zhang Q., Lin Y., Li J., Kang T., Tao M. (2014). Disrupting the Interaction of BRD4 with Di-acetylated Twist Suppresses Tumorigenesis in Basal-like Breast Cancer. Cancer Cell.

[71] Kim M.H., Kim J., Hong H., Lee S.-H., Lee J.-K., Jung E., Kim J. (2016). Actin remodeling confers BRAF inhibitor resistance to melanoma cells through YAP/TAZ activation. EMBO J..

[72] Nguyen C.D.K., Yi C. (2019). YAP/TAZ Signaling and Resistance to Cancer Therapy. Trends Cancer.

[73] Cheng H., Zhang Z., Rodriguez-Barrueco R., Borczuk A., Liu H., Yu J., Silva J.M., Cheng S.K., Perez-Soler R., Halmos B. (2015). Functional genomics screen identifies YAP1 as a key determinant to enhance treatment sensitivity in lung cancer cells. Oncotarget.

[74] Yu J., Chen L., Cui B., Wu C., Choi M.Y., Chen Y., Zhang L., Rassenti L.Z., Ii G.F.W., Kipps T.J. (2017). Cirmtuzumab inhibits Wnt5a-induced Rac1 activation in chronic lymphocytic leukemia treated with ibrutinib. Leukemia.

[75] Karvonen H., Perttilä R., Niininen W., Barker H., Ungureanu D. (2018). Targeting Wnt signaling pseudokinases in hematological cancers. Eur. J. Haematol..

[76] Qi J., Li X., Peng H., Cook E.M., Dadashian E.L., Wiestner A., Park H., Rader C. (2018). Potent and selective antitumor activity of a T cell-engaging bispecific antibody targeting a membrane-proximal epitope of ROR1. Proc. Natl. Acad. Sci. USA..

[77] Liu X., Pu W., He H., Fan X., Zheng Y., Zhou J.-K., Ma R., He J., Zheng Y., Wu K. (2019). Novel ROR1 inhibitor ARI-1 suppresses the development of non-small cell lung cancer. Cancer Lett..

